# Observations on the Practice of the Late Mr. James Ware, in Applying Vinous Tincture of Opium in Some Cases of Ophthalmia

**Published:** 1815-07

**Authors:** Joseph Brown

**Affiliations:** Queen's-Head Lane, Islington


*For the Medical and Physical Journal.*

### *Observations on the Practice of the late Mr. James Ware, in applying Vinous Tincture of Opium in some Cases of Ophthalmia;* by J. Brown, M. D.

IN the obituary of that popular and scientific miscellany, the Monthly Magazine, which was published the first day of this month, p. 360, is given a brief account of that late worthy man and successful oculist James Ware, Esq. The writer says, that "he was the first professor who applied laudanum topically in cases of inflammation of the eye, and recommended it for general practice, in a pamphlet that attracted much notice at the time; but, after the experience of many years, finding it injurious rather than beneficial, he had the manliness and candour to acknowledge that he had adopted an erroneous system, and wholly relinquished it."

This information, which, I believe, appeared also in the newspapers, I presume to think is not correct, because, more than thirty years ago, my left eye being painful and inflamed, I went to my worthy and intelligent friend Mr. Jonathan Wathen, surgeon, in Bond-court, Walbrook, who, after examining my eyes, and asking a few questions, rang the bell, and desired Mr. Ware, who was then his assistant, to be requested to come to us in the parlour, and bring with him the brown drop bottle. When Mr. Ware joined us, he was desired to instil into my afflicted eye two or three drops of Tinctura thebaica, which being done, Mr. Ware retired.

As soon as the smarting occasioned by the tincture subsided, Mr. Wathen told me, that, from the success with which he used liquid laudanum, to remove certain morbid affections of the eye, he expected that mine would soon be well. By the next day I was much better, that I repeated my visit, when Mr. Wathen himself repeated the process; and, in the course of our conversation, I observed that some antient medical writers encouraged the faculty to expect, in certain cases, the most beneficial effects to follow the external use of anodynes, a practice that, for cogent reasons, has of late years become very prevalent.

Messrs. Wathen and Ware were at one time in partnership: after they separated, Mr. Ware frequently appeared in print; print; therefore it is not unlikely that he was the first person who imparted to the public the use of laudanum in the manner above mentioned. Hence, the late lamented Mr. Saunders, and other writers on diseases of the eye, generally give Mr. Ware the credit of this method of treating chronic and strumous ophthalmia.

When the College of Physicians in London reformed their Pharmacopœia in 1788, they inserted a formula for a spirituous, and omitted the vinous, tincture of opium. Mr. Ware afterwards publicly noticed his preference of the latter: there is not, I am confident, any just reason to believe that he "relinquished" its use; on the contrary, I have had frequent experience of its utility, and I know the practice is continued by surgeons whose skill in their profession has never been doubted; and the Vinum Opii was inserted in the last London Dispensatory.

In one of the late Mr. Ware's publications, he recommended Pulvis Hydrarg. vitriolati. compositus, to be taken as snuff, for curing gutta serena. When I first read that essay, I instantly recollected that I had seen, in the 3d edition of a book, published, I believe, in 1710, a similar recipe. The ingenious compiler of Pharmacopœia Chirurgica, and other writers, constantly connect the name of Mr. Ware, exclusively of all others, with that formula, although Pulvis Hydrarg. comp. was recommended by Dr. Radcliffe; by Dr. Strother, see his Pulvis Ptarmicus (forte) 1719. Turbith mineral was also recommended by Dr. Brooke, see Ophthalmia, p. 381 of his Dispensatory, 3d edition, 1763; and subsequently by Dr. Hope, Professor of Botany at Edinburgh, who said, before the year 1789, that "he found the *turbith mineral* the most convenient errhine he had occasion to employ." Medical writers, in general, were, formerly, very often remiss in their duty, by not giving with precision directions concerning the doses of the medicines they employed: some merit is due to Mr. Ware for being more explicit. JOSEPH BROWN.

*Queen's-head Lane, Islington;*
*May* 10, 1815.

